# Cancer vaccines: the importance of targeting oncogenic drivers and the utility of combinations with immune checkpoint inhibitors

**DOI:** 10.18632/oncotarget.27861

**Published:** 2021-01-05

**Authors:** Erika J. Crosby, H. Kim Lyerly, Zachary C. Hartman

**Keywords:** breast cancer, tumor immunology animal models, T cell exhaustion, immune responses to cancer, tumor antigens/neoantigens

Evidence continues to accumulate supporting the viability of therapeutic cancer vaccines. It is now widely accepted that engagement of the adaptive immune system is a positive prognostic indicator for many types of cancer. The presence of cytotoxic T cells in tumors, engagement of CD4 T cell help, induction of tumor specific antibodies, and systemic engagement of antigen presentation and activation are all recognized as critical factors in an effective anti-tumor immune response [1, 2]. The use of a therapeutic agent like a vaccine targeting cancer antigens is an effective way to re-engage both arms of the adaptive immune response to overcome the inherent central and peripheral immune tolerance that exists against ‘self’ [3]. Ideally, vaccination will present antigens in tumor draining lymph nodes to initiate and support a durable anti-tumor response by both T cells and B cells, which can then circulate to the site of tumors to engage and destroy tumor cells. This process can further prime the immune response, as additional antigens are released from dying cancer cells and presented in secondary lymphoid organs [1]. Despite this relatively straightforward premise, the complexity of the process has led to many questions regarding the best antigen targets, vectors, timing, and combinations to optimize cancer vaccine therapeutic efficacy.

Cancer vaccines targeting HER2 provide a well-known example of a targeting strategy against a clinically credentialed tumor antigen that is known to play a key role in tumorigenesis, and has been successfully targeted by HER2-specific antibodies. The HER2-specific monoclonal antibodies (mAb) trastuzumab and pertuzumab are approved as part of a regimen with chemotherapy in the metastatic, neoadjuvant, and adjuvant settings that has become standard of care for HER2-positive breast cancer and has improved clinical outcomes of women with HER2 overexpressing breast cancer [4]. While the therapeutic activity of trastuzumab was originally ascribed to the inhibition of intracellular signaling, a growing body of evidence has demonstrated that engagement of the immune system is the likely mechanism for the therapeutic effects of trastuzumab and potentially other monoclonal antibodies *in vivo* [5]. A recent study suggests that this efficacy may be critically dependent upon innate immune engagement of macrophages through antibody-dependent phagocytosis, without a significant effect on adaptive immune responses [6]. As such, a vaccine strategy that not only engages T cells, but also broadens the endogenous anti-HER2 antibody response by increasing recognized epitopes and antibody elicited effector functions (such as antibody dependent phagocytosis, cytotoxicity and complement activation) has the potential to limit immune escape and resistance.

While a variety of vaccines targeting HER2 have been developed (based on proteins, peptides, modified tumor cells, viral vectors, plasmid DNA, and dendritic cells (DC)), none to date have been able to demonstrate broad clinical efficacy. Nonetheless, results from phase I and II studies of HER2-targeting cancer vaccines have revealed that HER2 is immunogenic, and that adaptive immune responses against HER2 may be associated with an improved clinical outcome [7–11]. These studies are also congruent with earlier observations that demonstrate an association between clinical outcome and tumor infiltrating T cells, immune signatures, and HER2-specific immune responses in patients [12–14]. Collectively, these clinical HER2 vaccination studies suggest that multiple types of HER2-specific vaccines share a common favorable safety profile. Many vaccines demonstrate the ability to elicit detectable circulating immunologic responses and some report favorable clinical outcomes. However, they also demonstrate that the elevated level of immunosuppression in advanced cancers will pose a significant challenge to achieving adaptive immune responses with therapeutic efficacy simply by vaccination alone. This begs the question, how can we optimize the immunogenicity and efficacy of cancer vaccines, as well as positively influence immune effector engagement within different types of immunosuppressive tumor microenvironments?

A successful vaccine must often overcome four major obstacles: (1) low immunogenicity of many cancer antigens; (2) immune tolerance to self (or largely self) antigens (3) established disease burden; and (4) the immunosuppressive tumor microenvironment (TME). In our recent work, we utilize a spontaneous model of HER2-driven breast cancer that expresses GFP in tumor cells. This system allowed us to ask a very basic question about cancer vaccine development. Would targeting a tumor driver self-antigen, such as HER2, be preferable to targeting a bystander, tumor-associated antigen (TAA), like GFP? Our studies of different models revealed that vaccination against GFP had no real impact on tumor progression, despite generating anti-GFP adaptive immune responses [15]. In contrast, targeting the oncogenic tumor driver HER2 through different means and models was consistently able to slow tumor growth through stimulation of HER2-specific adaptive immunity. Our use of a viral HER2-specific vaccine stimulated the most robust anti-tumor effect, overcoming immune tolerance to a self-antigen in an endogenous model of HER2+ breast cancer. However, this effect was related to tumor stage and greatly diminished if the established tumor burden was too high when the vaccine was administered. Thus, while the vaccine alone was very successful at inducing HER2-specific T and B cells when given early in the course of disease, no complete tumor regressions were seen when mice were vaccinated with more advanced cancers. As such, efforts to improve upon vaccinations through utilizing alternative vectors, heterologous prime boosting, as well as altered antigen targeting may permit more potent and efficacious vaccines to function against advanced cancers. These improvements will allow a more potent ability to break immune tolerance against tumor specific antigens, as well as result in a better quantity and quality of adaptive immune response, as we have demonstrated against HER2 in transgenic mice using a lysosomal targeting strategy [16]. However, even improved vaccines will likely have limitations and may not be able to overcome the immunosuppressive TME present in many advanced tumors. Thus, we speculated that an immune checkpoint inhibitor (ICI) might mitigate this immunosuppression and synergize with a HER2-specific vaccine.

ICI therapies have revolutionized cancer immunotherapy by addressing one of the biggest issues facing the field- broad immunosuppression due to chronic antigen stimulation by largely self-antigens. These highly conserved checkpoints serve to limit immunopathology during infection but have been co-opted by tumors to undercut a productive anti-tumor immune response. While ICIs have been highly successful for a subset of patients, most patients see limited benefit to these drugs as monotherapies [17]. Seemingly countless combination therapies are currently being tested, but one of the most promising is the pairing of an immune stimulating cancer vaccine with an ICI to mitigate the existing immunosuppression within the TME. While our HER2-targeting viral vaccine was able to slow tumor progression, a combination therapy of HER2 viral vaccine and anti-PD-1 ICI led to long-term survival of approximately 30% of treated animals [15]. Using single-cell RNA sequencing of T cells isolated from treated tumors, we were able to demonstrate the expansion of HER2-specific CD8+ T cells within the tumor microenvironment by the vaccine that had a highly activated gene expression profile. This was present regardless of treatment with anti-PD-1 ICI. However, the addition of anti-PD-1 ICI completely abrogated a potent exhaustion gene signature present in tumor-infiltrating T cells following vaccine alone. Clinically, these promising studies have led to the initiation of a phase II clinical trial testing a similar novel HER2 vaccine in combination with pembrolizumab (NCT03632941) to determine whether this approach can elicit effective anti-tumor immunity while minimizing off-target immune responses in patients with advanced HER2+ breast cancer.

While this approach provides evidence for the utility of combination therapies, the redundancy of the immune system ensures that a subset of patients will not respond or will progress. Indeed, while PD-L1 expression was highly elevated in nearly all tumors, long-term survival responses from the combination only occurred in 30% of mice [15]. Thus, alternative types of ICI will likely play a critical role in the activation of T cell responses in many cancers. T cell targeted ICIs are now used as single agents or in combination with chemotherapy as first or second lines of treatment for about 50 cancer types, with more than 3000 active clinical trials evaluating T cells modulators, representing about 2/3 of all oncology trials [17, 18]. Additionally, there has been renewed emphasis on alternative ICIs targeting innate immune cells to enable the functionality of tumor-specific antibodies (such as the CD47-SIRPα axis) [6], which may also play a key role in enabling adaptive immune responses elicited through vaccination. Indeed, early clinical indications of CD47 blockade suggest significant potential to enhance antibody-based cancer therapeutics [19]. However, understanding the optimal combinations to utilize in specific cancers, as well as their appropriate sequencing in combination with vaccination will be essential to making clinical progress in this area. But given the scope of advances in vaccine technology in combination with the revolution in immune checkpoint inhibition, there is good reason to be optimistic.
